# The use of latent variable mixture models to identify invariant items in test construction

**DOI:** 10.1007/s11136-017-1680-8

**Published:** 2017-08-23

**Authors:** Richard Sawatzky, Lara B. Russell, Tolulope T. Sajobi, Lisa M. Lix, Jacek Kopec, Bruno D. Zumbo

**Affiliations:** 1 School of Nursing, Trinity Western University, 7600 Glover Rd, Langley, BC V2Y1Y1 Canada; 2 Centre for Health Evaluation and Outcome Sciences, Providence Health Care, Vancouver, BC Canada; 3 Department of Community Health Sciences & O’Brien Institute for Public Health, University of Calgary, Calgary, AB Canada; 4 Department of Community Health Sciences, University of Manitoba, Winnipeg, MB Canada; 5 School of Population and Public Health, University of British Columbia, Vancouver, BC Canada; 6 Arthritis Research Canada, Vancouver, BC Canada; 7 Measurement, Evaluation & Research Methodology Program, University of British Columbia, Vancouver, BC Canada

**Keywords:** Measurement invariance, Latent variable mixture models, Test construction, Differential item functioning

## Abstract

**Purpose:**

Patient-reported outcome measures (PROMs) are frequently used in heterogeneous patient populations. PROM scores may lead to biased inferences when sources of heterogeneity (e.g., gender, ethnicity, and social factors) are ignored. Latent variable mixture models (LVMMs) can be used to examine measurement invariance (MI) when sources of heterogeneity in the population are not known a priori. The goal of this article is to discuss the use of LVMMs to identify invariant items within the context of test construction.

**Methods:**

The Draper-Lindely-de Finetti (DLD) framework for the measurement of latent variables provides a theoretical context for the use of LVMMs to identify the most invariant items in test construction. In an expository analysis using 39 items measuring daily activities, LVMMs were conducted to compare 1- and 2-class item response theory models (IRT). If the 2-class model had better fit, item-level logistic regression differential item functioning (DIF) analyses were conducted to identify items that were not invariant. These items were removed and LVMMs and DIF testing repeated until all remaining items showed MI.

**Results:**

The 39 items had an essentially unidimensional measurement structure. However, a 1-class IRT model resulted in many statistically significant bivariate residuals, indicating suboptimal fit due to remaining local dependence. A 2-class LVMM had better fit. Through subsequent rounds of LVMMs and DIF testing, nine items were identified as being most invariant.

**Conclusions:**

The DLD framework and the use of LVMMs have significant potential for advancing theoretical developments and research on item selection and the development of PROMs for heterogeneous populations.

## Introduction

Factor analysis and item response theory (IRT) methods are established methods for item selection in test construction for quality of life and patient-reported outcomes measures (PROMs) [1]. These methods focus on the dimensionality of a set of candidate items, where the goal is to identify those items that conform to a hypothesized and theoretically defensible dimensional structure. Measurement invariance is another important psychometric criterion that pertains to the equivalence of measurement model parameters across different subgroups of people in the population. This is particularly important when instruments are to be used in potentially heterogeneous populations of people who may differ in how they interpret and respond to questions about their health and quality of life. If the differences are caused by factors that are unrelated to the construct of interest, a test (i.e., measurement instrument) may produce biased scores. For example, if some respondents provide lower ratings for a general health item because they have difficulty in reading and understanding the item, their scores will be influenced by literacy, whereas the scores of others who have no difficulty in reading and understanding the item will not. This may in turn lead to incorrect inferences about the meaning of the scores, which are assumed to reflect only the construct of interest.

Several authors have argued for the importance of examining measurement invariance in test construction [2–5]. However, a particular challenge during test construction is that it is often not known a priori what characteristics of a population result in a lack of measurement invariance. In those situations, conventional approaches for examining measurement invariance with respect to selected manifest variables [6] will be of limited use. Latent variable mixture models (LVMMs) have been proposed to address this challenge; they can be used to examine measurement invariance with respect to two or more latent (i.e., unobserved) classes [7–9].

In this paper, we propose and describe the use of LVMMs to guide the identification of invariant items in test construction. We first introduce the Draper-Lindley-de Finetti (DLD) framework of latent variable measurement as a useful theoretical context [10, 11]. We then discuss how LVMMs could be used to assess measurement invariance. The methodological approach for using LVMMs in the context of test construction is discussed next. This is followed by a brief expository analysis demonstrating the approach using an existing item bank for the measurement of daily activities.

## Theoretical context

The DLD framework relates the measurement of latent variables to two necessary conditions pertaining to the exchangeability of both measurement items and sampling units (i.e., people or groups of people) [10, 11]. The first condition is that the items must be exchangeable such that they covary in a manner that is congruent with the measurement structure. Here, exchangeability refers to the notion that the items of a test are assumed to be drawn from a hypothetical pool of all possible representative items measuring the construct of interest (i.e., their dependencies are due only to the construct). The second condition is that the sampling units in the target population must be exchangeable such that the measurement model parameters are equivalently applicable to all individuals. These conditions reflect the fundamental assumption of local independence [12, 13], which requires that (a) dimensionality among the items is accurately represented in the measurement structure, *and* (b) item responses provided by individuals, or groups of individuals, are independent from those provided by other individuals in the target population. In other words, violations of local independence may be due to heterogeneity among the items or heterogeneity within the sample [14].

The DLD framework further relates the conditions of exchangeability of items and sampling units to the types of inferences that can be made based on test (e.g., PROM) scores [11]. In so doing, it provides an important basis for measurement validation, where the focus is on the validity of inferences (including actions and decisions) that are made on test scores [15]. The particular inferences of interest here pertain to the extent to which a pool of items consistently reflects a latent variable in a potentially heterogeneous population. *Exchangeability of items *is necessary to warrant inferences about the test scores irrespective of the combination of items that are administered. In the DLD framework, this is referred to as “specific domain inference” [11], which is particularly important when there are different versions of a measurement instrument (e.g., short forms) or when people are exposed to different measurement items (e.g., in computerized adaptive testing). *Exchangeability of sampling units* refers to the homogeneity of the population. This condition is necessary to warrant “specific sampling inference” [11] based on a measurement structure and estimated parameters that are equivalently applicable (i.e., invariant) across different subgroups in the population.

A variety of statistical methods are available for examining each condition. The first condition, *exchangeability of items*, relates to the dimensional structure of a set of measurement items. Unidimensionality implies that the items are exchangeable with respect to a single latent variable; that is, their covariances are fully accounted for by the latent variable. Factor analysis and item response theory (IRT) methods are widely used to evaluate this condition during the process of test construction [1, 16]. Items that conform to a hypothesized and theoretically defensible dimensional structure are retained, while those that do not (e.g., do exhibit small factor loadings or discrimination parameters, cross-loadings on other dimensions, poor internal consistency reliability, etc.) may be removed or revised, unless there are other reasons for retention.

The second condition, *exchangeability of sampling units*, relates to the degree to which residual covariances among items are explained by differences among individuals within the sample. Differential item functioning (DIF) methods are used to examine this condition by determining the invariance of item parameters with respect to various observed groups in the target population, such as those characterized by differences in demographic variables (gender, age, ethnicity) or various health-related variables (e.g., having one or more medical conditions). Examples of DIF techniques include multigroup confirmatory factor analysis [17, 18], the Mantel–Haenszel procedure [19], logistic regression models [20–22], multidimensionality-based procedures [23] such as the simultaneous item bias test (SIBTEST) [24], and IRT DIF analysis techniques [25–28]. In summary, the DLD framework provides a useful theoretical context for test construction by drawing our attention to statistical conditions focusing on exchangeability of both items and sampling units. A predominant focus in test construction has been on the exchangeability of items by examining dependencies among items to inform item selection. The DLD framework provides the rationale for also focusing on the exchangeability of sampling units by considering the extent to which the measurement model parameters of individual items are equivalent, or invariant, across population subgroups. If the goal is to construct a measure that is broadly applicable in a general population, it is important to identify those items for which the parameters are most invariant. However, a limitation of conventional DIF techniques for the assessment of measurement invariance is that the relevant sources of DIF in the target population must be known a priori [14, 29–31]. As a result, DIF analyses will only be as good as the selection of observed variables that represent sources of DIF, which are unlikely to fully capture population heterogeneity [29, 30]. This limitation is of particular concern when measurement instruments are used in large and potentially heterogeneous populations where the measurement model parameters are assumed to be invariant irrespective of *any* differences, known and unknown, in the target population. LVMMs are increasingly recommended to address this limitation by examining measurement invariance with respect to subgroups that are not specified a priori [7, 11, 14, 31–33].

## LVMMs for examining measurement invariance

LVMMs allow for the simultaneous modeling of continuous latent variables that represent dependencies among measurement items (exchangeability of items), and latent classes that accommodate dependencies among individuals (exchangeability of sampling units). The latent classes represent subgroups of people who, relative to the overall population, are more homogeneous with respect to a specified statistical model (e.g., a measurement model). LVMMs have been used for a number of purposes, including, for example, to identify groups of individuals who exhibit certain response behaviors (e.g., socially desirable responding [e.g., 34], test taking behaviors [e.g., 35]). They have also been used to identify groups of individuals with different symptom patterns and characteristics related to psychological conditions, such as anxiety sensitivity, panic disorder, and conduct disorder [e.g., 36–39], and have been proposed as a tool in the development of diagnostic classifications [e.g., 40]. In the context of test development, our interest lies in the use of LVMMs for the assessment of measurement invariance. Here, the focus is on measurement structures that include a continuous latent variable representing the construct of interest and latent classes (subgroups of individuals) that are defined by differences in the parameter estimates of the latent variable. If these differences occur between classes of individuals who are matched on the construct of interest, there is evidence that the measure lacks invariance.

Various LVMMs have been proposed for examining measurement invariance, including factor mixture models, Rasch and IRT mixture models, and extensions thereof. Factor mixture models combine factor analysis with latent class analysis by “nesting” the latent factor model within two or more latent classes [41–43]. In factor analysis, the measurement structure is assumed to hold across the population of interest. The addition of latent classes relaxes this assumption by allowing measurement model parameters (factor loadings, items thresholds or intercepts, and item residual variances) to vary across the classes. Similarly, in Rasch and IRT mixture models, the assumption of parameter invariance can be relaxed and population heterogeneity accommodated by allowing difficulty and discrimination parameters to differ across latent classes [29, 30, 44]. Based on these foundations, LVMMs can be used for the identification of invariant items in the context of test construction.

## LVMM approach for item selection

**Fig. 1:**
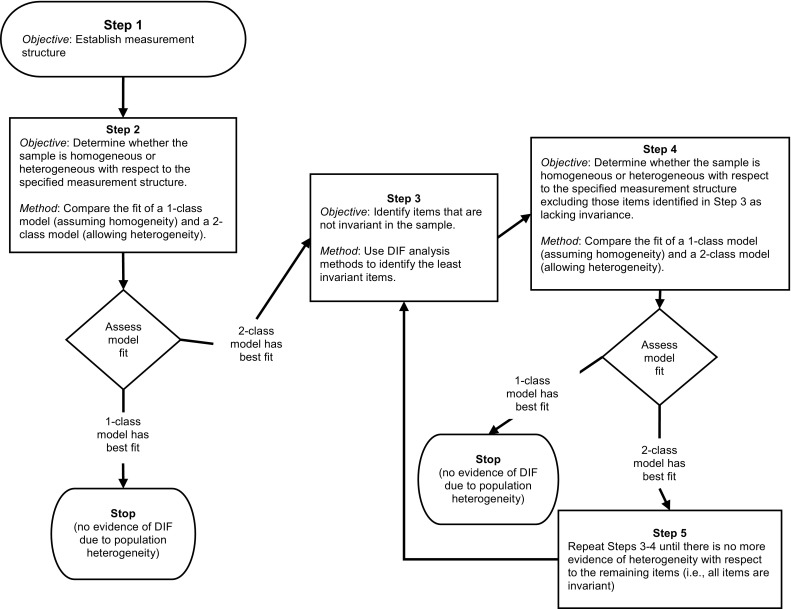
Analytic Approach for Using LVMMs in Test Construction. This figure is illustrative of comparing 1- and 2-class models. If considering more than 2 latent classes, Step 2 is expanded to sequentially decide on the number of latent classes, *k*, where *k* is greater than 2. Once the *k* classes are decided upon, the remaining steps are adapted to accommodate the *k*-class solution

The assessment of measurement invariance in the context of test construction comprises the following five sequential steps of identifying and removing noninvariant items while comparing the fit of resulting 1- and 2-latent class models (see Fig. 1). The approach can be described as follows (methodological details are presented in the expository analysis):

- Step 1: The first step pertains to the exchangeability of items, where the objective is to establish a theoretically defensible measurement structure of a candidate pool of items through the application of factor analysis methods in the full sample.
- Step 2: The next step is to determine whether a sample is homogeneous or heterogeneous relative to the measurement structure. This is accomplished by fitting the model from Step 1 to the data in both 1- and 2-class LVMMs and comparing the fit of the models. If the fit of the 1-class model is superior, there is no evidence of sample heterogeneity with respect to the measurement structure, and the measurement invariance analyses can be stopped. If the 2-class model produces better fit, the next step is to identify the items that contribute to this heterogeneity (i.e., the items that are least invariant).^1^
- Step 3: DIF methods are applied to identify those items that lack measurement invariance across the latent classes. These items are then removed from the test or item set (unless there are other reasons for retaining them).
- Step 4: The reduced test or item set is once again fit to the data in both 1- and 2-class LVMMs, and the fit of the models is compared. If the 1-class model produces better fit, the analyses come to an end. If the 2-class model produces better fit, an iterative process begins.
- Step 5: Steps 3 and 4 are repeated until the most invariant items are identified and the 1-class model produces superior fit compared with the 2-class model (i.e., the sample is no longer heterogeneous with respect to the measurement model).

It is important to note that the above steps focus on the identification of items for which the measurement model parameters are most likely to be invariant. In the context of test construction, this information supplements other psychometric and substantive considerations to guide item selection.

## Demonstration of LVMMs in test construction

The following expository analysis is provided as an example of how LVMMs can be used to identify items that are invariant in the population. The five-step approach was applied to an existing item bank (39 items) measuring daily activities (see Table 1), which is one of the item banks of the CAT-5D-QOL [45, 46]. The items address overall ability to perform usual activities, difficulty or limitations in specific aspects of daily living (e.g., grooming, working, and socializing) and the need for assistance in daily living. Five-point response scales were used for 37 items, while a 4-point and a 3-point response scale were used for one item each. The data are from a sample of 1666 adults living in the province of British Columbia, Canada. Approximately 20% were patients at a rheumatology clinic, 20% were drawn from a waiting list for knee or hip replacement surgery, and the remainder comprised a random stratified community sample. Further information about this sample is published elsewhere [8].

**Table 1:** DIF analysis results for models based on 39 and 9 items

Item	Item content (# response options)	Δ*R* ^2a^	
		39 items	9 items
100	Difficulty preparing one’s own meals (5)	Invariant	Invariant
105	Difficulty grooming oneself (5)	Invariant	Invariant
109	Difficulty using the toilet (5)	Invariant	Invariant
112	Difficulty performing light household chores (5)	Invariant	Invariant
123	Difficulty bathing oneself without help (5)	Invariant	Invariant
124	Difficulty dressing and undressing oneself (5)	Invariant	Invariant
128	Need for help with using the toilet (5)	Invariant	Invariant
130	Need for help with getting around the house (5)	Invariant	Invariant
137	Ability to take care of oneself (3)	Invariant	Invariant
111	Difficulty socializing with family and friends outside the home (5)	Invariant	0.207
114	Difficulty participating fully in social or family life (5)	Invariant	0.182
119	Difficulty socializing with family and friends inside the home (5)	Invariant	0.144
98	Limitations in usual social activities with family or friends (5)	Invariant	0.136
127	Need for help with getting dressed and undressed (5)	Invariant	0.056
115	Difficulty participating in nonphysical leisure activities (5)	Invariant	0.041
125	Need for help with bathing (5)	Invariant	0.039
110	Difficulty participating with enjoyment in strenuous leisure activities (5)	0.326	
104	Limitations in participation in strenuous leisure activities (5)	0.250	
116	Limitations in participation in physical leisure activities (5)	0.177	
118	Limitations in ability to perform heavy household chores (5)	0.161	
108	Difficulty participating in physical leisure activities (5)	0.160	
131	Difficulty performing heavy household chores (5)	0.159	
117	Difficulty accomplishing more than usual in work, school or other activities (5)	0.115	
120	Difficulty accomplishing as much as usual in work, school or other activities (5)	0.090	
99	Difficulty going shopping for groceries (5)	0.089	
102	Limitations in accomplishing more than usual in work, school or other activities (5)	0.087	
107	Difficulty performing normal work or other daily activities (5)	0.086	
122	Limitations in doing work as carefully and accurately as usual (5)	0.086	
103	Difficulty doing work as carefully and accurately as usual (5)	0.080	
97	Difficulty doing daily work (5)	0.077	
132	Difficulty getting in and out of a car (5)	0.075	
126	Difficulty washing face and hands (5)	0.073	
133	Difficulty traveling around the neighborhood without help (5)	0.070	
95	Problems with daily activities (general) (4)	0.070	
96	Overall ability to perform usual daily activities (e.g., work, leisure, self-care) (5)	0.052	
113	Difficulty getting around the house (5)	0.050	
129	Need for help with eating meals (5)	0.048	
101	Difficulty feeding oneself (5)	0.045	
106	Limitations in social activities with family or friends (5)	0.036	

^*a*^Δ*R* ^2^ is the difference in the Nagelkerke *R* ^2^ of model (i), with each item regressed on the factor score, and model (ii), where each item is regressed on the factor score, latent class membership, and their interaction. Invariant items are those that have a Δ*R* ^2^ < 0.035

### Statistical methods

The statistical methods of relevance to this expository analysis include those pertaining to factor analysis, IRT, LVMMs (using the MPLUS v7.4 software [47]), and DIF analysis (using SPSS v24 [48]).

For step 1, confirmatory and exploratory factor analyses were conducted using mean and variance weighted least squares estimation (WLSMV) to determine if the items could be treated as unidimensional. Dimensionality was assessed by evaluating the ratio of the first and second eigen values. Although the eigen value greater than 1 rule-of-thumb is widely used, it is important to note that it tends to result in overestimation of the number of latent factors [49, 50]. Based on a simulation study of conventional guidelines, Slocum-Gori and Zumbo recommend that a ratio of the first and second eigen values greater than 3 is indicative of a unidimensional structure when samples are relatively large (of 800 or more) and communality relatively high (the simulation was based on a communality of 0.90) [51]. Fit of the measurement model was assessed using the Comparative Fit Index (CFI) and Root Mean Square Error of Approximation (RMSEA). Values above 0.90 for the CFI and below 0.08 for the RMSEA indicate acceptable fit [52]. Next, a 2-parameter graded response IRT model using full information maximum likelihood was applied [53].

For step 2, LVMMs of the graded response IRT model from step 1 were applied specifying 1 and 2 latent classes, following model specifications described by Sawatzky et al. [7]. Relative fit of the 1- and 2-class LVMMs was assessed based on the Bayesian Information Criterion (BIC). Lower BIC values indicate better fit [54]. In addition, the percentage of statistically significant bivariate residuals (based on a *χ* ^2^ test of each item pair adjusted for multiple comparisons) was considered, as was the entropy for the 2-class model. Statistically significant bivariate residuals indicate violations of the assumption of local (item) independence [12, 13], while entropy measures certainty in class membership (values above 0.8 are considered indicative of high confidence in assignment) [55]. The assumed standard normal distributions of the latent factors were examined by describing the distributions of the predicted latent factor scores. Multinomial logistic regression based on pseudo-class draws [56, 57] was used to determine the extent to which latent classes differed with respect to sex, age, having a medical condition (yes/no), using two or more medications (yes/no), hospitalization during the previous year (yes/no), and self-reported health status (ranging from 1 = excellent to 5 = very poor).

For step 3, any of the aforementioned DIF methods could be used to examine measurement invariance of item parameters across the latent classes. For this expository analysis, the ordinal logistical regression (OLR) approach was used [22, 58]. This was accomplished by comparing two nested models where each item was regressed on (i) the latent factor score (based on the LVMM) and (ii) the factor score plus the latent class membership (to test for uniform DIF) and the latent class by latent factor interaction (to test for nonuniform DIF). The magnitude of DIF was evaluated based on the difference in the Nagelkerke *R* ^2^ (i.e., Δ*R* ^2^), comparing models (i) to (ii), for each item. A Δ*R* ^2^ below .035 is indicative of “negligible” DIF, a Δ*R* ^2^ between .035 and .070 indicates “moderate” DIF, and a Δ*R* ^2^ above .070 indicates “large” DIF [59]. Based on these criteria, the least invariant items were identified as those that had a Δ*R* ^2^ greater than .035.

For step 4, the 2-parameter graded response IRT model from Step 1, minus the least invariant items from Step 3, was refit to the data in both 1- and 2-class LVMMs. Model fit was assessed as in Step 2. In step 5, steps 3 and 4 were repeated several times, each time removing the items that exceeded the Δ*R* ^2^ cut-off.

## Results

Information about the fit of the LVMMs is reported in Table 2. The following is a summary of the results pertaining to each step of the LVMM approach.

**Table 2:** Model fit and latent-class estimation for latent variable mixture models

	Estimated model					
	39 items		16 items		9 items	
	1-class model	2-class model	1-class model	2-class model	1-class model	2-class model
BIC	77000	75287	27268	26781	13862	13942
Latent factor distribution						
Mean	0.00	0.01	0.00	0.00	0.00	0.00
Standard deviation	0.97	0.95	0.92	0.87	0.85	0.76
Skewness	−0.04	0.18	0.40	0.75	0.88	0.88
Kurtosis	−0.80	−0.75	−0.92	−0.48	−0.37	−0.37
Test of bivariate residuals						
# of item pairs	741	741	120	120	36	36
% <0.05^a^	45.2	9.5	30.0	5.0	8.3	0.0
Entropy	–	0.85	–	0.71	–	0.66
Class proportions						
Class 1	–	0.64	–	0.74	–	0.75
Class 2	–	0.36	–	0.26	–	0.25

^a^Adjusted for multiple comparisons using Bonferroni correction (corresponding with the number of item pairs being tested)

- Step 1: The EFA results produced a ratio of the first and second eigen values of 16.6, with the first four eigen values being 31.09, 1.87, 0.99, and 0.65, thereby providing support for unidimensionality. The single-factor structure resulted in acceptable overall model fit (RMSEA = .082; CFI = .986) and large standardized factor loadings, ranging from 0.76 to 0.96, with a median of .90 and an interquartile range from .88 to .94. However, 8.5% of the residual polychoric correlations are greater than 0.1 (the largest residual correlation is 1.78), which indicates areas of remaining local dependence. Having the compelling evidence of a unidimensional structure, we proceeded with examining heterogeneity in the population as an alternative explanation for the remaining local dependence.
- Step 2: The 2-class LVMM provided a better fit to the data compared with a 1-class graded response IRT model. The BIC for the 2-class LVMM was lower, and there was a notable reduction in the percentage of statistically significant bivariate residuals (see Table 2). The entropy for the 2-class LVMM was 0.84. The predicted latent factor scores of both models approximated the normal distribution (see Table 2). People in class 1 were more likely to be older, female, and have more health challenges (see Table 3). Because these results are suggestive of heterogeneity in the sample with respect to the measurement model, the next step was the identification of DIF items.

**Table 3:** Describing latent classes

Variables	Full sample	Model with 39-items			Model with 16 items		
		Class 1	Class 2	OR (95% CI)^a^	Class 1	Class 2	OR (95% CI)^a^
Sex (% female vs. male)	60.7	66.0	58.5	1.38 (1.09–1.74)	59.2	64.7	1.26 (0.97–1.65)
Age (mean (SD) in years)	56.7 (15.9)	61.9 (15.6)	54.6 (16.1)	1.03 (1.02–1.04)	56.5 (17.0)	56.7 (16.9)	1.00 (0.99–1.01)
Taking medications							
% None (referent)	22.3	5.4	28.9		12.7	25.8	
% 1 medication	23.5	13.7	27.3	2.66 (1.59–4.49)	20.5	24.6	1.70 (1.08–2.67)
% 2 medications	54.2	80.9	43.8	9.83 (6.19–15.6)	66.8	49.6	2.75 (1.87–4.06)
Hospitalized during past year (% yes)^b^	27.6	32.9	15.7	2.63 (2.02–3.41)	26.3	18.5	1.58 (1.18–2.10)
Treatment for rheumatoid arthritis (% yes)^b^	28.3	50.5	19.2	4.29 (3.38–5.45)	32.5	26.4	1.34 (1.03–1.76)
Treatment for osteo-arthritis (% yes)^b^	38.2	56.7	28.8	3.23 (2.56–4.07)	47.2	32.8	1.83 (1.42–2.36)
Has another health condition (% yes)^b, c^	74.0	86.9	73.6	2.39 (1.75–3.26)	84.7	71.2	2.25 (1.38–3.68)
Self-reported health during past 4 weeks (% “fair or poor”)^d^	24.0	41.5	17.2	3.43 (2.67–4.40)	38.6	18.8	2.72 (2.07–3.56)

^a^ *OR* unadjusted odds ratios based on binary logistic regressions with pseudo-class draws (referent = class 2), *CI* 95% confidence intervals

^b^Referent = no

^c^Reports having one or more of the following conditions: heart disease, high blood pressure, lung disease, diabetes, ulcer or stomach disease, kidney disease, liver disease, anemia or blood disease, cancer, depression, back pain, other medical problem

^d^Referent = good, very good, or excellent
- Step 3: OLR revealed that of the 39 items, 23 items had Δ*R* ^2^ values exceeding the recommended cut-off (see Table 1). These were removed from the model, and the resulting 16-item model was retested in Step 4.
- Step 4: A comparison of the 1- and 2-class LVMMs of the 16 items indicated that the 2-class model once again had better fit (see Table 2). The two classes differed with respect to several demographic- and health-related variables (see Table 3).
- Step 5: OLR (next iteration of step 3) was subsequently reapplied to the remaining 16 items based on the LVMM results from step 4. Five items had Δ*R* ^2^ values above the recommended cut-off and were removed. The BIC of the 1-class LVMM of the remaining nine items was lower than that of the 2-class LVMM (next iteration of step 4). In addition, the 1-class LVMM of the remaining nine items resulted in substantially improved fit relative to the 1-class LVMMs of 16 and 39 items. These results suggest that the sample is relatively more homogeneous with respect to the unidimensional measurement structure of the nine items. Therefore, no further DIF analyses were conducted.

A factor analysis of the final selection of nine items provided compelling support for a unidimensional measurement structure (the two largest eigenvalues were 7.5 and 0.4) and similar overall model fit (RMSEA = 0.087; CFI = 0.99), and substantially improved local independence, with only one residual correlation above 0.1 (*r* = 0.11). The parameter estimates of the corresponding unidimensional graded response model are reported in Table 4. Finally, the predicted factors scores are strongly correlated with the factor scores (*r* = 0.83) based on a graded response model of the original 39 items.

**Table 4:** Parameter estimates of the 9-item graded response model^a^

Item	*λ* (SE)	*τ*_1_ (SE)	*τ*_2_ (SE)	*τ*_3_ (SE)	*τ*_4_ (SE)
Q100	4.49 (0.48)	3.92 (0.45)	5.55 (0.54)	7.56 (0.67)	9.76 (0.88)
Q105	4.68 (0.62)	5.45 (0.66)	7.57 (0.75)	9.99 (1.01)	14.85 (1.77)
Q109	3.77 (0.64)	5.34 (0.92)	7.04 (0.88)	8.98 (1.06)	10.62 (1.11)
Q112	5.31 (0.66)	4.90 (0.60)	7.40 (0.77)	9.13 (0.90)	11.97 (1.24)
Q123	4.65 (0.80)	6.10 (1.08)	7.55 (1.07)	8.87 (1.20)	9.75 (1.25)
Q124	6.96 (1.30)	9.51 (2.00)	12.13 (2.13)	14.70 (2.64)	16.37 (2.52)
Q128	4.10 (0.94)	9.23 (1.70)	10.02 (1.78)	10.76 (1.92)	11.70 (2.01)
Q130	2.90 (0.37)	5.46 (0.50)	6.48 (0.54)	7.56 (0.64)	8.15 (0.72)
Q137	5.58 (1.19)	9.10 (1.78)	13.39 (2.55)		

^a^The parameters are of a mixture graded response model as specified in the MPlus [47] software where the cumulative probability *Ρ*_*ij*_ of an item *i* response at or above category *j* is expressed as follows: $P_{ij} \left( {Y \ge j|\theta } \right) = \frac{{\exp ( - \tau_{ij} + \lambda_{i} \theta )}}{{1 + \exp ( - \tau_{ij} + \lambda_{i} \theta )}},$ where *τ*_*ij*_ denotes the thresholds between the categories of item *i*, and *λ*_*i*_ denotes the factor loading for item *i*. The following transformation can be applied to convert the Mplus thresholds (*τ*) and factor loadings (*λ*) into the difficulty (*β*) and discrimination (*α*) parameters of the graded response model: $\beta_{ij} = \frac{{\tau_{ij} }}{{\lambda_{i} }},$ and $\alpha_{i} = \lambda_{i}$

## Discussion

Factor analysis methods are widely used to guide item selection in test construction. The DLD framework provides a theoretical basis for examining measurement invariance as an additionally important consideration. However, the characteristics of individuals that may affect measurement invariance are often not known a priori. For example, DIF analyses could have been conducted based on the subsamples in the data used for our expository analysis (rheumatology patients, hip and knee patients, and a community sample). While this approach might also lead to the detection of DIF items, and would be appropriate if the goal were to establish lack of DIF relative to these groups specifically, such a manifest groups approach would fail to detect DIF with respect to the more complex set of characteristics that describe the latent classes found in our data (Table 3). Although others have advocated for consideration of measurement invariance in test construction, this is the first study to describe and demonstrate how LVMMs can be used to identify invariant items to inform item selection in the development of PROMs.

In our expository analysis, we used LVMMs to identify a subset of items that were most invariant within the sample. We specifically demonstrate how LVMMs can complement IRT analysis to examine and address the assumption of local independence underlying latent variable measurement theory. As aptly described in the DLD framework, local independence requires exchangeability of items (dimensionality) as well as of sampling units (invariance) [10, 11]. However, despite the apparent utility of LVMMs to inform item selection based on the exchangeability of sampling units, these models do not always provide conclusive results. Accordingly, it is widely acknowledged that item selection should not be exclusively driven by these statistical considerations. Both item content and theoretical considerations need to be taken into account [2, 16]. For example, in our analysis, most of the retained items address difficulty related to basic activities of daily living at more severe levels of disability (e.g., dressing, bathing, toilet etc.), whereas items pertaining to social activities and leisure activities were not retained. Consequently, content validity, and therefore construct validity, may have been affected by the removal of items. Further validation research is needed to determine the extent to which the remaining items fully reflect the intended construct of interest. The estimated correlation of the factor scores based on the original 39 items and the remaining 9 items is quite large (i.e., 0.83), providing support for concurrent validity. However, the correlation is not perfect. Depending on the purpose of measurement and the conceptualization of daily activities, different decisions about the retention of items, or the option of revising items to be more invariant, may be made.

There are several important areas for further methodological development regarding the use of LVMM for the identification of least invariant items. First, simulation studies are recommended to determine the optimal sequential process for removing items that lack measurement invariance. In the example analysis, all items that met a particular criterion for invariance were removed before refitting the LVMM. The rationale is to remove those items that lack invariance with respect to particular latent classes, prior to estimating new latent class parameters. Another option is to remove one item at a time, such that the latent class parameters are reestimated every time an item is removed. Second, as is common in factor analysis, IRT, and Rasch analysis, the LVMMs in our analysis assume normally distributed latent factors (although this is not a necessary condition for latent variable modeling). LVMMs may detect artefactual latent classes when this assumption is not met [60]. In addition, although the widely used graded response model was used in our analysis, other IRT and Rasch models could be utilized. Simulation studies are needed to determine the extent to which mis-specification of latent factor distributions and different specifications of latent variable measurement structures may affect LVMM results in the context of test construction. Third, simulation studies are recommended for determining the potential implications of multidimensionality with respect to identification of DIF and the use of LVMMs, for “[a]lthough the presence of DIF automatically implies the presence of a secondary dimension, the presence of a secondary dimension does *not* automatically imply the presence of DIF” [61, p. 108]. While our expository analyses exemplifies the application of LVMM to a unidimensional set of items, it is important to consider the challenges of distinguishing multidimensional constructs from DIF, especially when there is evidence of “nuisance dimensions”, which could be manifestations of DIF [24, 29, 62]. Fourth, it is not known to what extent DIF analyses may be influenced by inconclusive class membership (i.e., entropy values less than 1). In addition, other DIF detection methods and effect size criteria for identifying invariant items could be utilized [6]. The OLR DIF detection approach utilized in the expository analysis was chosen because it is relatively straightforward to conduct and has a strong track record in psychometric analyses of PROMs. Although extensive research comparing different DIF detection methods has been conducted [e.g., 6], previous studies have not focused on the application of these methods in relation to LVMMs. Simulation studies and primary research can be used to develop specific recommendations for implementing LVMMs across a range of data-analytic conditions.

## Conclusion

We propose a theoretical foundation and general approach for using LVMMs in test construction with the intent to stimulate further methodological development for heterogeneous populations. An important goal in the measurement of PROMs is to ensure that the perspectives of patients are represented in an unbiased manner. The DLD framework and use of LVMMs have significant potential for advancing theoretical developments and research on item selection for test construction of PROMs in heterogeneous populations.

## Footnotes

1 It is possible to fit models with additional latent classes in an incremental stepwise fashion, and this may in fact better capture any existing heterogeneity in the sample. However, it should be noted that the introduction of additional classes can significantly increase the complexity of the DIF analyses, as many more parameters and models need to be compared. For this reason, we have limited our expository analysis to the comparison of 1- and 2-class LVMMs.
